# Marker-Assisted Selection to Pyramid the *Opaque-2 (O2)* and β-Carotene *(crtRB1)* Genes in Maize

**DOI:** 10.3389/fgene.2019.00859

**Published:** 2019-09-26

**Authors:** Sarankumar Chandran, Bharathi Pukalenthy, Karthikeyan Adhimoolam, Dhasarathan Manickam, Vellaikumar Sampathrajan, Vanniarajan Chocklingam, Kokiladevi Eswaran, Kavithapushpam Arunachalam, Laishram Joikumar meetei, Ravikesavan Rajasekaran, Vignesh Muthusamy, Firoz Hossain, Senthil Natesan

**Affiliations:** ^1^Department of Plant Breeding and Genetics, Agricultural College and Research Institute, Tamil Nadu Agricultural University, Madurai, India; ^2^Department of Biotechnology, Agricultural College and Research Institute, Tamil Nadu Agricultural University, Madurai, India; ^3^Department of Plant Biotechnology, Center for Plant Molecular Biology and Biotechnology, Tamil Nadu Agricultural University, Coimbatore, India; ^4^Department of Soil Science and Agricultural Chemistry, Agricultural College and Research Institute, Tamil Nadu Agricultural University, Killikulam, India; ^5^Department of Tree Improvement and Plant Breeding and Genetics, Central Agricultural University, Imphal, India; ^6^Department of Millet, Center for Plant Breeding and Genetics, Tamil Nadu Agricultural University, Coimbatore, India; ^7^Division of Genetics, ICAR—Indian Agricultural Research Institute, New Delhi, India; ^8^Department of Plant Molecular Biology and Bioinformatics, Center for Plant Molecular Biology and Biotechnology, Tamil Nadu Agricultural University, Coimbatore, India

**Keywords:** β-carotene, *crtRB1*, marker-assisted backcross breeding, *opaque-2*, quality protein maize

## Abstract

Maize is an excellent nutritional source and is consumed as a staple food in different parts of the world, including India. Developing a maize genotype with a combination of higher lysine and tryptophan, along with β-carotene, can help alleviate the problem of protein-energy malnutrition (PEM) and vitamin A deficiency (VAD). This study is aimed at improving lysine and tryptophan content by transferring *opaque-2* (*o2*) gene from donor HKI163 to β-*carotene*-rich inbred lines *viz*., UMI1200β^+^ and UMI1230β^+^. For this purpose, F_1_, BC_1_F_1_, BC_2_F_1_, BC_2_F_2_, and BC_2_F_3_ plants were developed using an o2 line HKI163 and two β-carotene-rich inbred lines, UMI1200β^+^ and UMI1230β^+^, as the parents. Foreground selection using the associated marker umc1066 for the *o2* gene and the marker *crtRB1* 3′TE for the *crtRB1* gene was used to select the target genes. A total of 236 simple sequence repeat (SSR) markers distributed evenly across the maize genome were employed for the background selection. To fix the *crtRB1* allele in the BC_1_F_1_ stage, individual plants homozygous at the *crtRB1* locus and heterozygous at the *o2* locus were selected and used for backcrossing to produce BC_2_F_1_ plants. Furthermore, the selected heterozygous BC_2_F_1_ plants from both crosses were selfed to obtain the BC_2_F_2_ plants, which were then selected for the target gene and selfed to generate the BC_2_F_3_ lines. From each cross, five improved lines with homozygous marker alleles for the *crtRB1* and *o2* genes with a recurrent parent genome (RPG) recovery ranging from 86.75 to 91.21% in UMI1200β^+^×HKI163 and 80.00 to 90.08% in UMI1230β^+^×HKI163 were identified. The improved lines had good agronomic performance and possessed high lysine (0.294–0.332%), tryptophan (0.073–0.081%), and β-*carotene* (6.12–7.38 µg/g) content. These improved lines can be used as genetic resources for maize improvement.

## Introduction

Maize (*Zea mays* L.) is a staple food crop and currently grown in more than 150 countries, with a total harvest area of approximately 187 million hectares, producing 1138 million tonnes worldwide (FAOSTAT, 2018). It has good nutritional value, that is, 68.5% carbohydrates, 8% fat, 4% ash, 3% crude fiber, and 16.5% protein (Ullah et al., 2010). In addition, maize carotenoids contain both provitamin A (뗐α-carotene, β-carotene, and β-cryptoxanthin) and non-provitamin A (lutein and zeaxanthin) components. Maize, therefore, is of special importance for the nutrition of people from many countries in Africa, Asia, and Latin America, where protein-energy malnutrition (PEM) and vitamin A deficiency (VAD) affect more than a billion people. The demand for maize has steadily increased over the past decades and is expected to continue to rise in the forthcoming years, at least up until 2050 (Rosegrant et al., 2009). However, normal maize protein possesses low nutritional significance to humans because of very limited amounts of major amino acids, such as lysine (1.6–2.6%) and tryptophan (0.2–0.6%) (Moro et al., 1996), which is less than half of the recommended dose specified for human nutrition. Over the past three decades, many natural maize mutants associated to quality protein maize (QPM) with higher lysine and tryptophan content have been identified, that is, *opaque-2 (o2)* in chromosome 7 (Mertz et al., 1964), *floury-2 (fl2)* in chromosome 8 (Nelson et al., 1965), *opaque-7 (o7)* in chromosome 8 (Ma and Nelson, 1975), *opaque-6 (o6)* in chromosome 8 (McWhirter, 1971), and *floury-3 (fl3)* in chromosome 8 (Ma and Nelson, 1975). Among them, *o2* mutant has been more popular and widely utilized in breeding programs for the improvement of protein quality. The recessive *o2* allele improves the endosperm lysine and tryptophan levels by nearly two-fold. The gene-linked simple sequence repeat (SSR) markers umc1066, phi112, and phi057 have been used to identify the *o2* gene (Yang et al., 2005; Gupta et al., 2013; Surender et al., 2017).

VAD is one of the serious health issues in developing and low-income countries and critically affects over 7 million pregnant women and 125 million children (Giuliano, 2017). *β-Carotene* is the best provitamin A (vitamin A precursor), and maize is a predominant source of *β-carotene*; however, very few maize varieties are rich in β-carotene, and many exhibiting varieties are inherently deficient in β-carotene (Muthusamy et al., 2014). Yan et al. (2010) revealed that *crtRB1* is a major gene responsible for the β-carotene content in maize. This gene is positioned at chromosome 10 and encodes β-carotene hydroxylase, which is responsible for the biosynthesis of lycopene. Association mapping approach led to the identification of three polymorphisms, 5’TE (in the 5’-Untranslated Region), InDel4 (in the coding region), and 3’TE (spanning the sixth exon and 3’-Untranslated Region), in the *crtRB1* gene that were significantly influencing the β-carotene content. Since then, polymerase chain reaction (PCR)-based codominant markers were developed based on these polymorphisms, and these markers aided breeders to identify and develop higher β-carotene content lines using marker-assisted selection (MAS). Moreover, Yan et al. (2010) reported the 3’TE favorable allele (allele 1, 543 bp) that is responsible for reduced transcript expression of the gene associated with higher β-carotene content, with an average increase of 6.50 μg/g in the maize endosperm in comparison with the unfavorable allelic class. Recently, this allele-based marker was successfully used to detect the *crtRB1* gene in diverse maize lines (Muthusamy et al., 2014; Zunjare et al., 2018; Sagare et al., 2019).

To date, numerous maize hybrids with either provitamin A or QPM have been released and commercialized, but genotypes with both the nutritional traits are very limited. This situation necessitates developing maize genotypes with the combination of QPM and provitamin A. Our previous attempts have led to the development of two β-carotene-rich inbred lines *viz*., UMI1200β^+^ and UMI1230β^+^. In this study, our objective was aimed to introgress the *o2* gene from HKI163 into UMI1200β^+^ and UMI1230β^+^. We, therefore, applied marker-assisted backcross (MAB) breeding using gene-specific markers for foreground selection and polymorphic SSRs for background selection. Our goal was to obtain innovative breeding materials with high β-carotene, lysine, and tryptophan contents.

## Materials and Methods

### Plant Genetic Materials

HKI163 is an inbred line containing the opaqueness gene *(o2)*. Its grain lysine content is 0.340% in protein, and its tryptophan content is 0.082% in protein (Zunjare et al., 2018). It was obtained from Chaudhary Charan Singh Haryana Agricultural University, Uchani, India. UMI1200β^+^ and UMI1230β^+^ are improved inbred lines containing the β-carotene-associated gene *crtRB1*, with a grain lysine content of 0.130 and 0.150%, respectively, and tryptophan content of 0.024 and 0.029%, respectively. These β-carotene-rich inbred lines were developed by transferring *crtRB1* gene from donor HP46715 (CIMMYT, Mexico) to local popular inbred lines *viz*., UMI1200 and UMI1230. The β-carotene contents of UMI1200β^+^ and UMI1230β^+^ were 9.073 and 9.232 µg/g, respectively.

### Development of Backcross Progenies

**Figure 1:**
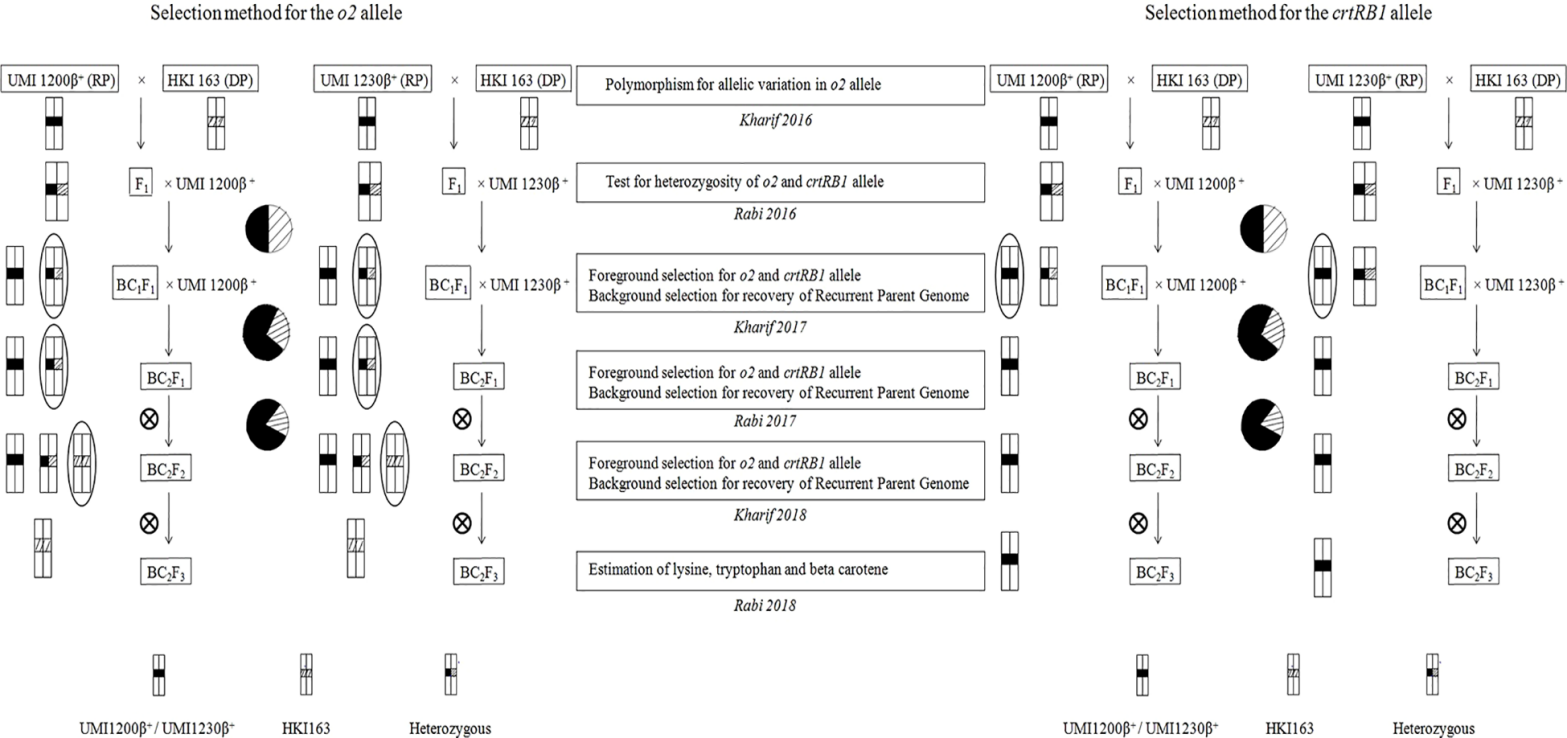
Scheme for the development of *o2* and *crtRB1* genes-derived improved lines using marker-assisted foreground and background selection.

MAB breeding scheme that includes crossing, backcrossing, and selfing was undertaken as mentioned in **Figure 1**. Backcross progenies were developed by crossing UMI1200β^+^ and UMI1230β^+^ (recurrent parents) with HKI163 (donor parent) following two cycles of backcrosses during 2016 to 2019. UMI1200β^+^ and UMI1230β^+^ were used as recurrent parents and crossed with HKI163 (donor) for developing F_1_ plants. Then, F_1_ plants were confirmed by foreground selection with *crtRB1* and *o2*-linked markers. These F_1_ plants were used as the male parents to develop the BC_1_F_1_s. Likewise, another round of backcross was followed for UMI1200β^+^×HKI163 and UMI1230β^+^×HKI163 to develop BC_2_F_1_s using MAB breeding to reduce the linkage drag and to increase the recurrent parent genome percentage. Furthermore, selected BC_2_F_1_ plants that were heterozygous at the *o2* loci and homozygous at the *crtRB1* loci were self-pollinated to produce BC_2_F_2_ plants and BC_2_F_3_ plants.

### Genomic DNA Isolation and PCR Analysis

Young leaf tissues from two-week-old plants were ground into powder using liquid nitrogen and stored at -80°C. Genomic DNA was isolated using the cetyl trimethylammonium bromide (CTAB) method (Murray and Thompson, 1980). The DNA was checked for its quantity and quality on a 0.8% agarose gel. The PCR for *crtRB1* 3′TE gene-specific and SSR primers and agarose gel electrophoresis were carried out following the method by Muthusamy et al. (2014) and Pukalenthy et al. (2019).

### Foreground and Background Selection

*o2* gene- and *crtRB1 gene*-linked markers were used for the foreground selection in backcross and selfed lines (**Table 1**). Based on marker polymorphism between donor and recurrent parents, three SSR markers, umc1066, phi 112, and phi057, linked to the *o2* gene and *crtRB1* 3′TE, which is linked to the *crtRB1*, were employed for foreground selection. For the background selection, a total of 236 SSR markers distributed on all 10 chromosomes of maize genome were used to identify polymorphic markers between the donor and recurrent parents. Furthermore, the SSR markers that showed polymorphism among the parents were used in the background selection to determine the recurrent parent genome (RPG) recovery percentage at each backcross generation. All of the SSR primer sequences used in background selection were obtained from the maize genome database (www.maizegdb.org) and synthesized by Eurofin Ltd, Bangalore, India.

**Table 1:** Sequence information of the markers used for polymorphic studies and foreground screening.

S. No.	Marker name	Forward sequence (5’-3’)	Reverse sequence (5’-3’)	Annealing
1	*phi112*	TGCCCTGCAGGTTCACATTGAGT	AGGAGTACGCTTGGATGCTCTTC	53°C
2	*umc1066*	ATGGAGCACGTCATCTCAATGG	AGCAGCAGCAACGTCTATGACACT	60°C
3	*phi 057*	CTCATCAGTGCCGTCGTCCAT	CAGTCGCAAGAAACCGTTGCC	63°C
4	*crtRB1*	ACACCACATGGACAAGTTCG	ACACTCTGGCCCATGAACAC (R1)	62°C -54°C^a^
			ACAGCAATACAGGGGACCAG (R2)	54°C

a In total of 19 cycles, reduction of temperature by 0.5°C on each cycle starting from the initial 54–62°C.

### Kernel Modification

The parents and heterozygous plant (*O2/o2*) seeds from backcrossed and selfed progenies (BC_1_F_1_, BC_2_F_1_, BC_2_F_2_, and BC_2_F_3_) were harvested and examined for the kernel modification using a standard light box screening method (Vasal et al., 1980). Maize kernels were categorized into five types *viz*., type 1, not opaque; type 2, 25% opaqueness; type 3, 50% opaqueness; type 4, 75% opaqueness; and type 5, 100% opaqueness (Vivek et al., 2008). In all of the generations, the kernels with 25% opaqueness were selected and forwarded to the next generation to fix the *o2* allele in its homozygous recessive state and to reduce the undesirable traits caused by the modifier genes acting in the maize endosperm.

### Investigation of Morphological Traits in Improved Lines

For the BC_2_F_3_ improved lines, observations for 15 morphological traits that were categorized and presented chronologically according to the plant stage data were taken using standard maize descriptors formulated by the International Board for Plant Genetic Resources (IBPGR) (Anonymous, 1991). Morphological traits *viz*., days to tasselling (days), days to silking (days), plant height (in centimeters), ear height (in centimeters), tassel length (in centimeters), number of tassel branches, leaf length (in centimeters) and leaf width (in centimeters), cob length (in centimeters), cob girth (in centimeters), number of kernel rows per cob, number of kernels per row, cob weight (in grams), single plant yield (in grams), and 100-kernel weight (in grams) were taken.

### Estimation of Lysine, Tryptophan, and *β*-Carotene Contents

The lysine, tryptophan, and β-carotene contents were estimated in seeds of BC_2_F_3_ improved lines. The shelled seeds taken for estimation were shade dried and stored at 22–26°C before the analysis. Lysine and tryptophan contents in the endosperm were estimated according to the method described by Galicia et al. (2008). The estimations were done with two replications consisting of two blanks, four checks, and the samples using the spectrophotometer V- 770 UV-VIS-NIT (Japan). The absorbances of lysine and tryptophan were recorded at 390 and 560 nm, respectively. The estimated lysine and tryptophan values were measured with the unit (in percent) (Moro et al., 1996). β-Carotene extraction was done according to the method described by Kurilich and Juvik (1999). The β-carotene content was estimated by high-performance liquid chromatography (HPLC), and samples were eluted by C30 column (5 μm, 4.6 × 250 mm). The mobile phase was composed of acetonitrile:dichloromethane:methanol (75:20:5). The retention and the spectrum of the carotenoid compounds were found to have a flow rate of 0.4 ml/min and were compared to those of the standard (β-carotene standard-M/s Sigma Aldrich, India). Furthermore, it was reconstituted in the acetonitrile mixture in three different concentrations (1, 10, and 100 ppm).

### Statistical Analysis

In BC_1_F_1_, BC_2_F_1_, and BC_2_F_2_ generations, the segregation distortion was studied by chi-square analysis for the deviation from the expected Mendelian ratio. In the background selection, the amplicons were scored as A for recurrent parent, B for donor parent, and H for heterozygous plants. The recovery percentage of the recurrent genome was calculated using the formula RPG (%) = [A + (0.5H)/(A + B + H)] × 100 (Benchimol et al., 2005).

## Results

### Development of Maize Inbred Lines With the *O2* and *crtRB1* Genes

**Figure 2:**
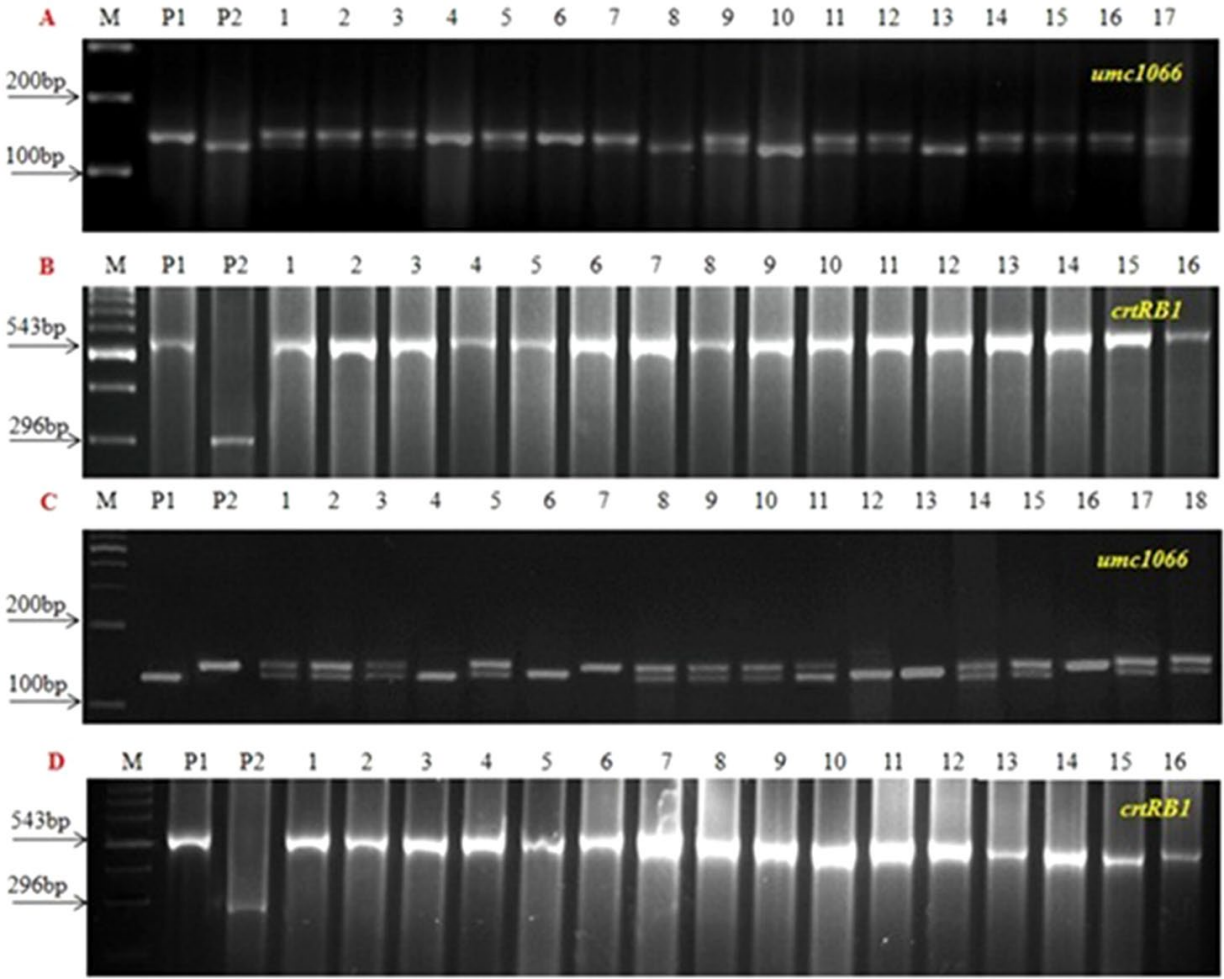
Foreground screening in BC_2_F_2_ progenies. **(A** and **B)** UMI1200β^+^xHKI163 **(C** and **D)** UMI1230β^+^xHKI163, (M) Marker 100bp, (P_1_) UMI1200β^+^/UMI1230β^+^, (P_2_) HKI163 and (1-16) BC_2_F_2_ progenies.

**Figure 3:**
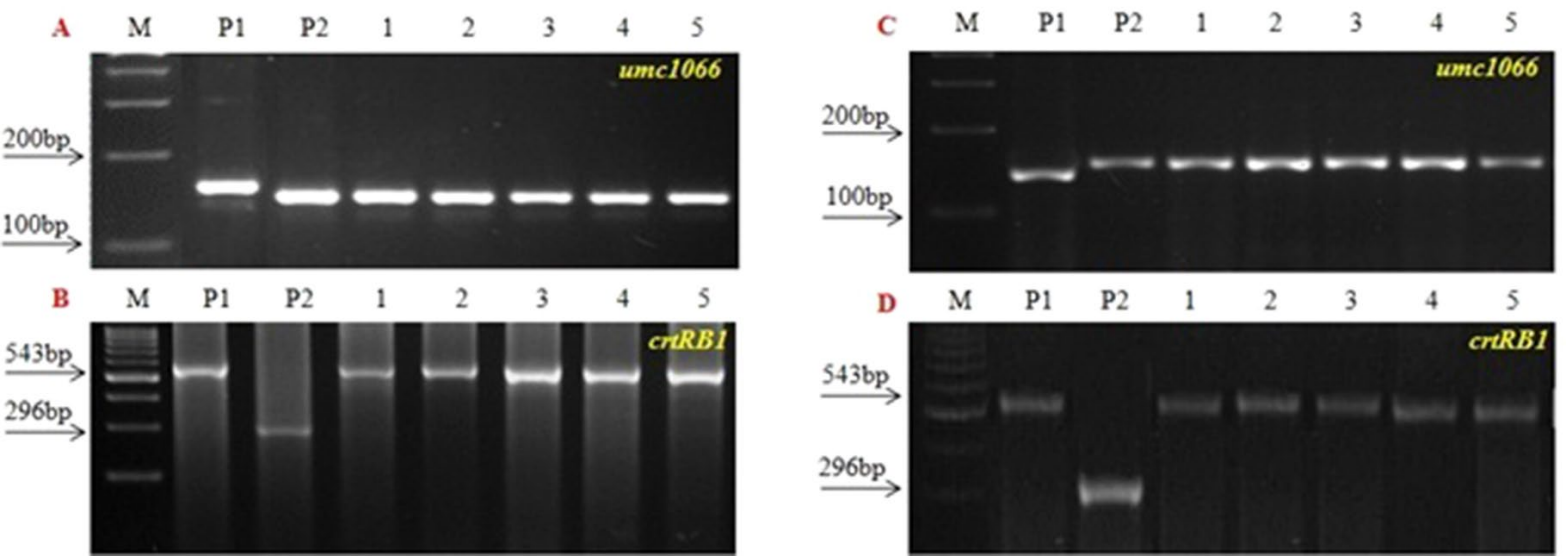
PCR screening of five improved BC2F3 lines using *o2* SSR and *crtRB1* gene specific marker. (A and B) UMI1200β^+^ × HKI163 and (C and D) UMI1230β^+^ × HKI163. (M) Marker 100 bp, (P1) UMI1200β^+^/UMI1230β^+^, (P2) HKI163. (1-5) Improved lines from UMI1200β^+^×HKI163 (1-DBT6-1-5/25-8/25-4/25-4/25, 2- DBT6-1-5/25-8/25-9/25-9/25, 3- DBT6-1-5/25-10/25-15/25-15/25, 4- DBT6-1-5/25-10/25-17/25-17/25, 5- DBT6-1-5/25-14/25-11/25-11/25) and UMI1230β^+^×HKI163 (1- DBT7-1-6/25-9/25-37/25-37/25, 2- DBT7-1-6/25-9/25-57/25-57/25, 3- DBT7-1-6/25-12/25-23/25-23/25, 4- DBT7-1-6/25-27/25-3/25-3/25, 5- DBT7-1-6/25-27/25-67/25-67/25).

Three SSR markers, umc1066, phi112, and phi057, located within the *o2* gene were investigated for their polymorphisms among the donor HKI163 and the two recurrent parents *viz*., UMI1200β^+^ and UMI1230β^+^. Among them, umc1066 was found to be polymorphic between the donor and each of the two recurrent parents. This informative SSR marker was further used for the foreground selection. F_1_ progenies were produced from two independent crosses of UMI1200β^+^×HKI163 and UMI1230β^+^×HKI163. BC_1_F_1_ progenies were obtained by backcrossing the F_1_ plants with UMI1200β^+^ and UMI1230β^+^ as the recurrent parents. In the BC_1_F_1_ generation, individual plants homozygous at the *crtRB1* and heterozygous at the *o2* locus were identified using the *crtRB1* and *o2*-gene specific markers and utilized for next backcrossing with the recurrent parent. Furthermore, BC_2_F_1_ progenies were obtained from the selected BC_1_F_1_ plants based on the dual-selection procedure involving foreground selection and light box screening. Applying similar selection procedures and selfing, progenies of BC_2_F_1_ generation were advanced to BC_2_F_2_ **(** **Figure 2**) and BC_2_F_3_. Finally, from each cross, five BC_2_F_3_ lines with homozygous marker alleles for the *CrtRB1* and *o2* genes were developed (**Figure 3**). The segregation patterns of backcross progenies are presented in **Table 2**.

**Table 2:** Segregation pattern of *o2* allele in the backcross and selfed generation of UMI1200β^+^×HKI163 and UMI1230β^+^×HKI163.

S. No.	Cross	Generation	No. of plants genotyped	Genotypic class			χ^2^	P value
				No. of A	No. of H	No. of B		
1	UMI1200β^+^ × HKI163	BC_1_F_1_	197	74	123	0	12.18782**	0.000481
		BC_2_F_1_	194	84	110	0	03.484536^ns^	0.061945
		BC_2_F_2_	126	33	082	11	19.143**	0.000069
2	UMI1230β^+^ × HKI163	BC_1_F_1_	175	40	135	0	51.5714286**	0.00006903
		BC_2_F_1_	100	43	057	0	01.96^ns^	0.161513
		BC_2_F_2_	106	36	040	30	07.566^ns^	0.0294

**Significant different (p < 0.01), the markers were significantly distorted from the expected segregation ratio (1:2:1); ^ns^non-significant (p ≥ 0.01), the markers were non-significant and fitted to the normal Mendalian ratio.

### SSR-Based Genetic Background Analysis of Improved Lines

**Figure 4:**
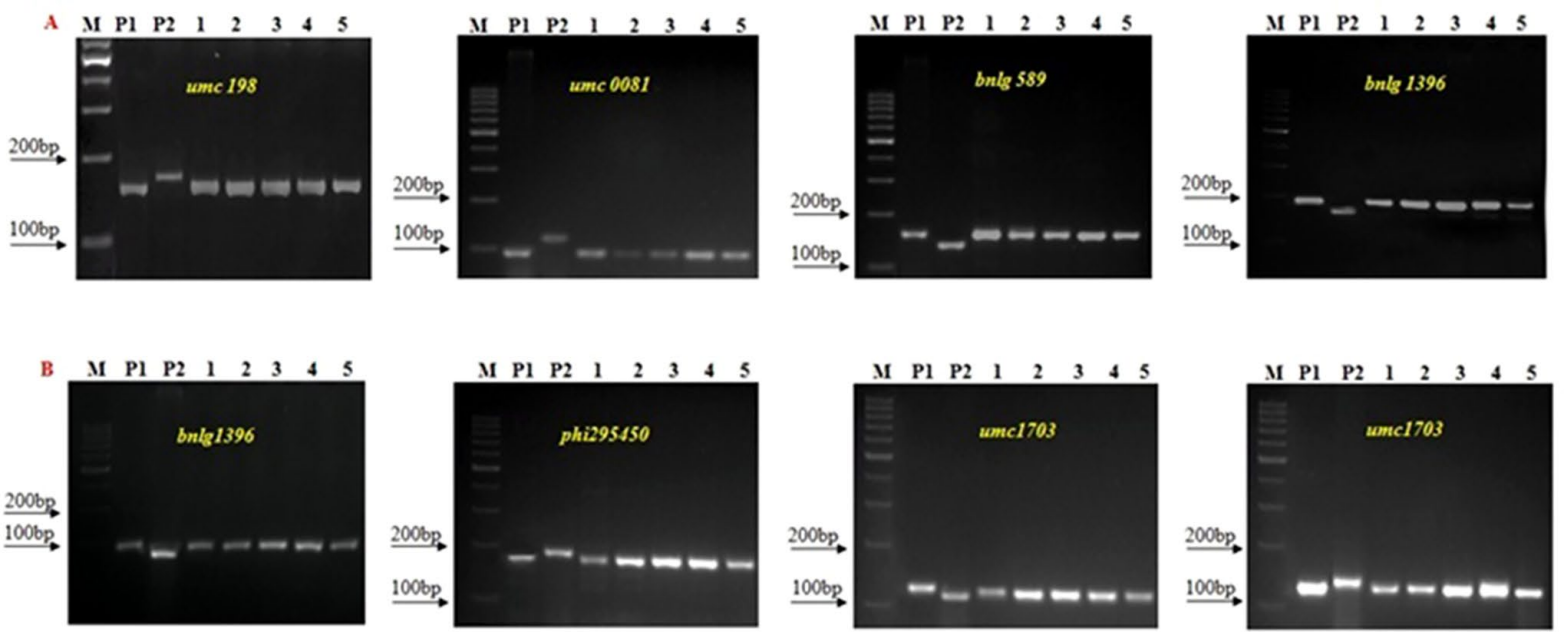
Background Screening of improved lines using SSR makers. **(A)** 1200β^+^xHKI163, **(B)** UMI1230β^+^xHKI163. (M) Marker 100bp, (P_1_) UMI 1200β^+^/UMI 1230β^+^, (P_2_) HKI163, (1-5) Improved lines from 1200β^+^xHKI163 (1-DBT6-1-5/25-8/25-4/25-4/25, 2-DBT6-1-5/25-8/25-9/25-9/25, 3- DBT6-1 5/25-10/25-15/25-15/25, 4-DBT6-1-5/25-10/25-17/25-17/25, 5- DBT6-1-5/25-14/25-11/25-11/-25) and 1230β^+^xHKI163(1- DBT7-1-6/25-9/25-37/25-37/25, 2-DBT6-1-6/25-9/25-57/25-57/25, 3- DBT7-1-6/25-12/25-23/25-23/25, 4- DBT7-1-6/24-27/25-3/25-3/25, 5-DBT7-1-6/25-27/25-67/25-67/25).

A set of 236 SSR markers distributed uniformly across the maize genome was used in polymorphism screening to select polymorphic markers between donor and recurrent parents. Among them, 104 and 107 SSR markers showed polymorphism between UMI1200β^+^ and HKI163 and UMI1230β^+^ and HKI163, respectively. The polymorphism percentage was recorded as 44.6 and 49.57%, respectively. Furthermore, these polymorphic markers were employed to screen the progenies derived from backcross and selfed generation for the recovery of RPG (**Figure 4**). In BC_1_F_1_ generation, a total of 22 and 18 foreground positive plants from UMI1200β^+^×HKI163 and UMI1230β^+^×HKI163 were screened, which showed a recovery of RPG of 54.81% and 53.21%, respectively. Furthermore, the recovery of RPG increased in subsequent generations. The 22 and 31 selected positive plants from UMI1200β^+^×HKI163 and UMI1230β^+^×HKI163 in BC_2_F_1_ showed 82.21 and 79.81%, of RPG, respectively. Eight and six positive plants from UMI1200β^+^×HKI163 and UMI1230β^+^×HKI163 in BC_2_F_2_ showed 87.48 and 86.51% recovery of RPG, respectively. The highest recoveries of RPG 91.21% and 90.08% were obtained in each of five BC_2_F_3_ plants from UMI1200β^+^×HKI163 and UMI1230β^+^×HKI163.

### Kernel Modification

**Figure 5:**
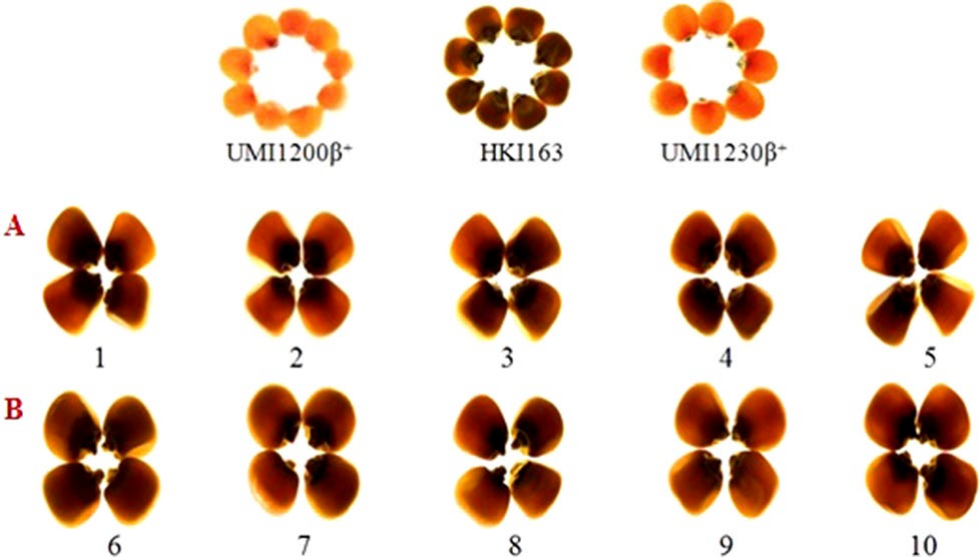
Endosperm modification of BC_2_F_3_ **(A** and **B)** of UMI1200β^+^xHKI163 and UMI1230β^+^xHKI163. **(A)** Five improved lines form the cross UMI1200β^+^xHKI163(1-DBT6-1-5/25-8/25-4/25-4/25, 2-DBT6-1-5/25-8/25-9/25-9/25, 3-DBT6-1-5/25-10/25-15/25-15/25, 4-DBT6-15/25-10/25-17/25-17/25, 5-DBT6-1-5/25-14/25-11/25-11/25) **(B)** Five improved lines from the cross UMI1230β^+^XHKI163(6-DBT7-1/25-9/25-37/25-37/25, 7-DBT7-1-6/25-9/25-57/25-57/25, 8-DBT7-1-6/25-12/25-23/25-23/25, 9-DBT7-1-6/25-27/25-3/25-3/25, 10-DBT7-1-6/25-27/25-67/25-67/25).

Opaqueness is the indicator for the presence of *o2* allele, it is also tightly linked to the *o2* gene, selecting the kernels along with the least opaqueness from generation to generation ensures that the *o2* gene is fixed in its homozygous recessive state. Thus, we observed the opaqueness in selected foreground positive progenies from backcrossed and selfed progenies, along with HKI163, UMI1200β^+^, and UMI1230β^+^ for kernel modification. HKI163 kernels showed 25 and 50% opaqueness, whereas UMI1200β^+^ and UMI1230β^+^ exhibited 0% opaqueness. BC_1_F_1_, BC_2_F_1_, and BC_2_F_2_ progenies showed 0–100% opaqueness. Among them, progenies showing 25% were further selected and advanced to next generation, whereas the remainder were rejected. In maize, the endosperm modifier genes play a major role to produce undesirable characteristics, which affect the crop yield. Thus, we selected the progenies with 25% opaqueness to reduce the effect of the *o2* modifier gene action. As a result, the recessive allele of *o2/o2* was fixed in maize kernels and all of the BC_2_F_3_ lines showed 25% opaqueness (**Figure 5**).

### Morphological Characteristics of Improved Lines

**Figure 6:**
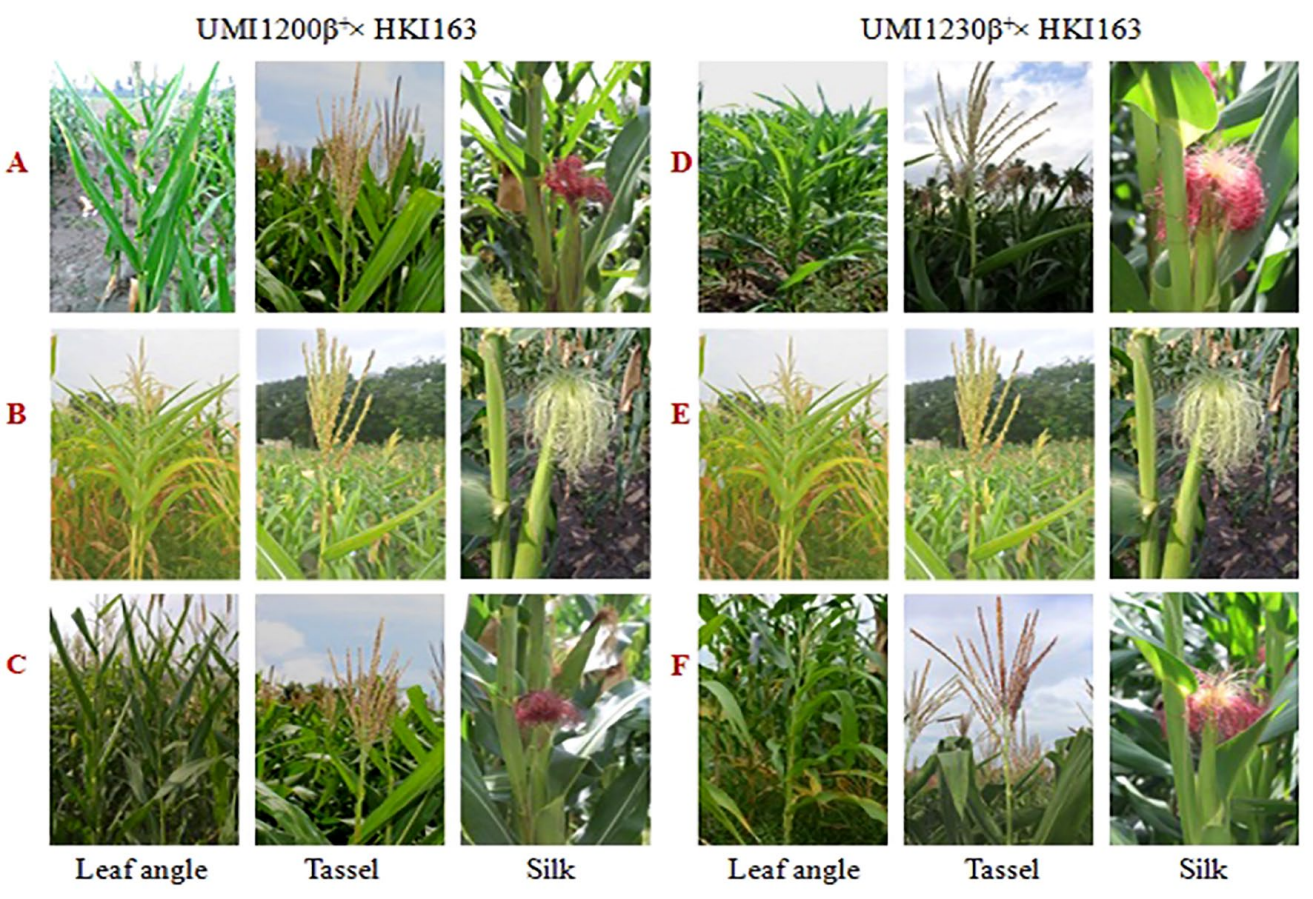
Morphological traits of parents and improved lines of UMI1200β^+^xHKI163 and UMI1230β^+^xHKI163. **(A)** UMI1200β^+^, **(B)** HK163, **(C)** DBT6-1-5/25-10/25-17/25- 17/25, **(D)** UMI1230β^+^, **(E)** HKI163 **(F)** DBT7-1-6/25-12/25-23/25-23/25.

The morphological traits of the improved lines along with their donor and recurrent parents were presented in **Table 3**. Five UMI1200β^+^×HKI163-based improved lines showed phenotypic resemblance ranging from 71.43% (number of kernels per row) to 98.38% (days to silking). Among them, DBT6-1-5/25-10/25-17/25-17/25 and DBT6-1-5/25-14/25-11/25-11/25 possessed high phenotypic resemblance with the recurrent parent. For instance, days to tasselling, days to silking, plant height, ear height, tassel length, cob girth, cob weight, 100-kernel weight, and single plant yield showed more than 90% similarity to UMI1200β^+^. Likewise, five UMI1230β^+^×HKI163-based improved lines showed phenotypic resemblance ranging from 82.43% (ear height) to 99.66% (100-kernel weight). DBT7-1-6/25-12/25-23/25-23/25 showed maximum similarity with the recurrent parent, followed by DBT7-1-6/25-27/25-67/25-67/25. These two lines exhibited more than 90% similarity to UMI1230β^+^ for the traits days to tasselling, days to silking, plant height, ear height, tassel length, leaf length, cob length, cob girth, cob weight, 100-kernel weight, and single plant yield (**Figure 6**).

**Table 3:** Comparison of BC_2_F_3_ improved lines from UMI1200β^+^×HKI163 and UMI1230β^+^×HKI163 with the parents for the recovery percentage of morphological traits.

Morphological traits	UMI1200β^+^ (Recurrent parent)	HKI163 (Donor parent)	Identified positive lines					Recovery percentage (%) for morphological trait				
(UMI1200β^+^ × HKI163)			DBT6-1-5/25-8/25-4/25-4/25	DBT6-1-5/25-8/25-9/25-9/25	DBT6-1-5/25-10/25-15/25-15/25	DBT6-1-5/25-10/25-17/25-17/25	DBT6-1-5/25-14/25-11/25-11/25	DBT6-1-5/25-8/25-4/25-4/25	DBT6-1-5/25-8/25-9/25-9/25	DBT6-1-5/25-10/25-15/25-15/25	DBT6-1-5/25-10/25-17/25-17/25	DBT6-1-5/25-14/25-11/25-11/25
Days to tasseling (days)	59.00	63.00	56.00	57.00	58.00	58.00	57.00	94.91	82.60	98.30	98.30	96.61
Days to silking (days)	62.00	65.00	58.00	60.00	61.00	60.00	59.00	93.54	96.77	98.38	96.77	95.16
Plant height (cm)	126.88	110.00	118.00	121.40	123.50	119.90	122.70	93.00	95.68	97.33	94.49	96.70
Ear height (cm)	71.00	62.00	68.00	67.70	69.30	68.30	68.00	95.77	95.35	97.60	96.19	95.77
Tassel length (cm)	21.00	26.20	18.00	19.20	18.10	19.50	20.30	85.71	91.42	86.19	92.38	96.66
Number of tassel branches	12.00	16.00	10.00	11.00	10.00	11.00	10.00	83.33	91.66	83.33	91.66	83.33
Leaf length (cm)	61.65	63.00	58.20	59.40	59.20	58.00	54.70	94.40	96.35	96.02	94.07	88.72
Leaf width (cm)	06.10	06.00	05.80	05.80	05.70	05.60	05.00	95.08	95.08	93.44	91.80	81.96
Cob length (cm)	15.20	17.00	13.00	13.80	14.00	13.00	12.40	85.52	90.78	92.10	85.52	81.57
Cob girth(cm)	13.80	13.00	12.00	11.50	13.00	12.70	13.10	86.95	83.33	94.20	92.02	94.92
Number of kernel rows per cob	14.00	12.00	12.00	10.00	12.00	10.00	10.00	85.71	85.71	71.43	85.71	71.43
Number of kernels per row	21.00	26.00	18.00	17.00	17.00	18.00	17.00	85.71	80.95	80.95	85.71	94.92
Cob weight (g)	108.00	112,00	98.00	92.80	94.30	101.00	97.90	90.74	85.92	87.31	93.51	90.64
100-kernel weight (g)	24.00	22.00	23.00	22.30	22.80	23.10	22.80	95.83	92.91	95.00	96.25	95.00
Single plant yield (g)	90.67	57.00	82.11	83.07	84.11	86.21	87.32	90.55	91.61	92.76	95.08	96.31
(UMI1200β^+^ × HKI163)	UMI1230β^+^ (Recurrent parent)	HKI163 (Donor parent)	DBT7-1-6/25-9/25-37/25-37/25	DBT7-1-6/25-9/25-57/25-57/25	DBT7-1-6/25-12/25-23/25-23/25	DBT7-1-6/25-27/25-3/25-3/25	DBT7-1-6/25-27/25-67/25-67/25	DBT7-1-6/25-9/25-37/25-37/25	DBT7-1-6/25-9/25-57/25-57/25	DBT7-1-6/25-12/25-23/25-23/25	DBT7-1-6/25-27/25-3/25-3/25	DBT7-1-6 /25-27/25-67/25-67/25
Days to tasseling (days)	61.00	63.00	58.00	58.00	59.00	60.00	58.00	95.08	95.08	96.72	98.36	95.08
Days to silking (days)	63.00	65.00	61.00	60.00	62.00	62.00	60.00	96.82	95.23	98.41	98.41	95.23
Plant height (cm)	112.00	110.00	110.50	109.70	104.60	101.00	105.20	98.66	97.94	93.39	90.17	93.92
Ear height (cm)	74.00	62.00	61.00	72.00	71.00	72.10	72.90	82.43	97.29	95.94	97.43	98.51
Tassel length (cm)	24.10	26.20	23.40	22.80	23.70	23.90	22.80	97.09	94.60	98.34	99.17	94.60
Number of tassel branches	14.00	16.00	13.00	12.00	13.00	12.00	12.00	92.85	85.71	92.85	85.71	85.71
Leaf length (cm)	61.00	63.00	58.40	60.20	59.00	59.20	58.90	95.73	98.68	96.72	97.04	96.55
Leaf width (cm)	06.50	06.00	06.30	6.10	05.70	05.80	05.90	96.92	93.84	87.69	89.23	90.76
Cob length (cm)	16.00	17.00	14.00	15.60	14.90	15.80	15.30	87.50	97.50	93.12	98.75	95.62
Cob girth (cm)	12.50	13.00	11.00	11.80	12.10	11.60	12.00	88.00	94.40	96.80	92.80	96.00
Number of kernel rows per cob	16.00	12.00	14.00	14.00	14.00	14.00	14.00	87.50	87.50	87.50	87.50	87.50
Number of kernels per row	23.00	26.00	22.00	22.00	22.00	21.00	19.00	95.65	95.65	95.65	91.30	82.60
Cob weight (g)	124.70	112.00	122.60	118.30	113.80	120.90	121.00	98.31	94.86	91.25	96.95	97.03
100-kernel weight (g)	29.50	22.00	27.40	28.10	28.90	29.40	28.70	92.88	95.25	97.96	99.66	97.28
Single plant yield (g)	76.98	57.00	72.11	69.43	71.67	72.11	73.31	93.67	90.19	93.10	93.67	95.23

### Analysis of Lysine, Tryptophan, and *β-Carotene*

All of the improved lines showed that lysine and tryptophan content increased many-fold over for both the β-carotene-rich parents *viz*., UMI1200β^+^ and UMI1230β^+^. Lysine and tryptophan contents varied from 0.294 to 0.332% and 0.073 to 0.081%, with an average of 0.314 and 0.077%. Among the improved lines, DBT 7-1-6/25-27/25-3/25-3/25 from UMI1230β^+^×HKI163 possessed higher levels of lysine (0.332%) and tryptophan (0.081%). Furthermore, accumulation of the β-carotene content was estimated in improved lines, which ranged from 6.127 to 7.387 µg/g with an average of 6.80 µg/g and higher than the QPM parent HKI163. DBT6-1-5/25-8/25-4/25-4/25 from UMI1200β^+^×HKI163 was found to have a high β-carotene content of 7.387 µg/g among improved lines. The lysine, tryptophan, and β-carotene contents of the improved lines are presented in **Table 4**.

**Table 4:** Lysine and tryptophan concentrations in parents and BC_2_F_3_ improved lines from UMI1200β^+^×HKI163 and UMI1230β^+^×HKI163.

Carotenoid/Amino acids	UMI1200β^+^	UMI1230β^+^	HKI163	(UMI1200β^+^ × HKI163)					(UMI1230β^+^ × HKI163)				
				DBT6-1-5/25-8/25-4/25-4/25	DBT6-1-5/25-8/25-9/25-9/25	DBT6-1-5/25-10/25-15/25-15/25	DBT6-1-5/25-10/25-17/25-17/25	DBT6-1-5/25-14/25-11/25-11/25	DBT7-1-6/25-9/25-37/25-37/25	DBT7-1-6/25-9/25-57/25-57/25	DBT7-1-6/25-12/25-23/25-23/25	DBT7-1-6/25-27/25-3/25-3/25	DBT7-1-6/25-27/25-67/25-67/25
β- carotene (µg/g)	9.073	9.232	0.800	7.387	6.127	6.665	7.187	6.865	7.123	7.265	6.321	6.812	6.312
Lysine (%)	0.130	0.150	0.340	0.294	0.312	0.299	0.322	0.331	0.298	0.317	0.308	0.332	0.327
Tryptophan (%)	0.024	0.029	0.082	0.077	0.079	0.080	0.073	0.080	0.073	0.077	0.079	0.081	0.075

## Discussion

### The Value of the Pyramiding *O2* and *crtRB1* Genes

Lysine, tryptophan, and β-carotene are the key nutritional traits in maize. The genetic nature and environmental factors have an influence on these traits. *crtRB1* and *o2* genes present on chromosomes 10 and 7 (Mertz et al., 1964; Vasal, 2000; Yang et al., 2004) provide increased β-carotene, lysine, and tryptophan contents. Molecular markers linked to these genes are available to facilitate direct selection in the breeding process. In this study, we successfully pyramided the *o2* and *crtRB1* genes in maize by MAS and several generations of backcrossing. The β-carotene content of the improved lines was increased by five- to six-fold for both crosses when compared to the QPM parent. The lysine and tryptophan contents of the improved lines were increased by two- and seven-fold for both crosses compared to the β-carotene parents. Thus, *o2* and *crtRB1* genes can work together in the same genetic background to control the content of lysine, tryptophan, and β-carotene.

### Development of Improved Lines Through MAB Breeding

Parental polymorphism screening revealed that recurrent parents UMI1200β^+^ and UMI1230β^+^ were clearly distinguishable with *o2* gene and *CrtRB1* gene*-*linked markers *umc1066* and *crtRB1* 3′TE from the donor line HKI163 and thus were used for foreground selection in the F_1_, BC_1_F_1_, BC_2_F_1,_ BC_2_F_2_, and BC_2_F_3_ generations. In foreground selection, F_1_ and BC_1_F_1_ generations screening with *crtRB1* allele indicated that all of the genotypes were heterozygous in the F_1_ generation and the segregation distortion in the BC_1_F_1_ generation (Babu et al., 2013). From the BC_1_F_1_ generation, the lines were fixed for the *crtRB1* allele by selecting the plants with favorable allele (543bp) and rejecting the heterozygous plants with both allele (543bp+296bp). Therefore, no segregation existed for *crtRB1* allele in the forwarded generations. Screening for the *o2* gene revealed that BC_2_F_1_ of UMI1200β^+^×HKI163 and BC_2_F_1_ and BC_2_F_2_ of UMI1230β^+^×HKI163 showed approximately 50% of heterozygous plants with respect to the expected Mendalian ratio (1:1) in the backcross generations and (1:2:1) in the selfed generations. However, segregation distortion was observed in BC_1_F_1_ and BC_2_F_2_ of UMI1200β^+^×HKI163 and BC_1_F_1_ of UMI1230β^+^×HKI163. These results are in accordance with the previous reports (Liu et al., 2015; Tripathy et al., 2017; Goswami et al., 2019; Sagare et al., 2019). Furthermore, background analysis using genome-wide SSR markers revealed 91.21 and 90.08% recovery of RPG in each of the five BC_2_F_3_ plants from UMI1200β^+^×HKI163 and UMI1230β^+^×HKI163 and coupled with the earlier findings (Feng et al., 2015; Sarika et al., 2018).

### Characteristics of Improved Lines

In addition to the background selection, phenotypic characterization is also useful to find the recovery percentage of recurrent parents (Manna et al., 2005; Gunjaca et al., 2008; Choudhary et al., 2014; Hossain et al., 2018). Phenotypic characterization among the parents and the improved lines showed more than 90% of recovery of the recurrent parents in morphological traits. Among them, DBT6-1-5/25-10/25-17/25-17/25 and DBT6-1-5/25-14/25-11/25-11/25 from UMI1200β^+^×HKI163 and DBT7-1-6/25-12/25-23/25-23/25 and DBT7-1-6/25-27/25-67/25-67/25 from UMI1230β^+^×HKI 163 possessed high phenotypic resemblance (90%) with their recurrent parents. Previously, several studies also reported more than 90% recovery of the recurrent parent characteristics in MAS-derived lines (Surender et al., 2017; Pukalenthy et al., 2019; Sagare et al., 2019). The lysine and tryptophan contents of the improved lines ranged from 0.294 to 0.331% and 0.073 to 0.080% for the cross UMI1200β^+^×HKI163 and 0.298 to 0.332% and 0.073 to 0.081% for the cross UMI1230β^+^×HKI163. On the average, lysine and tryptophan contents of the improved lines were 0.314 and 0.077%; they are at par with the QPM parent, three and seven-fold increases from the recurrent parents. Likewise, the average β-carotene contents of the improved lines for UMI1200β^+^×HKI163 and UMI1230β^+^×HKI163 were 6.846 and 6.766 µg/g, respectively, which were comparable to the β-carotene parents, six-fold higher than the QPM parent. Similar results were obtained by various studies (Muthusamy et al., 2014; Zunjare et al., 2018; Goswami et al., 2019). Overall, the improved inbred lines gained lysine and tryptophan contents but a slight reduction in β-carotene content (>2 ug) and grain yield. We followed the dual-selection procedure of molecular and light box screening to fix the *o2* allele, which is the reason behind increasing lysine and tryptophan contents. We selected the progenies based on the good agronomic performance (>90%) and β-carotene content, even though some of the progenies recorded β-carotene contents at par with the recurrent parents with less agronomic performance. Thus, a slight reduction was observed in β-carotene content (>2 ug) of improved inbred lines. Moreover, introgression of *o2* and *crtRB1* genes caused a reduction in the grain yield. It is reported that QPM lines have some undesirable characteristics because of the modifier gene action in the endosperm. Thus, we used dual selection procedure to select the progenies and developed the improved inbred lines with less undesirable traits along with *o2* and *crtRB1* genes. However, it is not possible to stop the modifier gene *(o2)* activity and remove the undesirable traits completely. It might influence the yield attributing traits and reduces the yield performances. Thus, we obtained a reduction in the grain yield similar to a previous study (Lauderdale, 2000).

In the present study, using MAB breeding approach, we successfully pyramided the *o2* and *crtRB1* genes and developed the nutrition-rich inbreds, but introgression of multiple genes caused a slight reduction in the yield. To utilize these newly developed inbred lines effectively, our future research focus is on conducting multilocation trial (MLT) in various maize-growing regions and identifying the superior inbred lines to develop new hybrids. In addition, these inbred lines can be used as genetic resources for maize biofortification programs.

## Data Availability

The datasets generated for this study are available on request to the corresponding author.

## Author Contributions

SN, FH, and VM designed the methods and experiments. SC, BP, DM, and RR developed backcross progenies and managed fieldwork. LJ and VC provided suggestions on experiments and monitored the work. SC, BP, DM, KAd, and KE conducted phenotype and genotype analysis. SC, VS, and KAr performed biochemical analysis. SC, BP, and DM analyzed the data. SC, KAd, and SN drafted the manuscript.

## Funding

Financial support of Department of Biotechnology (DBT), Government of India (GOI), through project entitled ‘Enrichment of nutritional quality in maize through molecular breeding’ (BT/PR10922AGII/106/9442014 dt.25.3.2015) and Ministry of Science and Technology, Department of Biotechnology, Government of India DBT’s Twinning program for the NE for the project entitled ‘Marker assisted introgression of LyCE gene for enhanced ProA in maize’ (BT/166/NE/TBP/2011 dt 12.12.2011) is acknowledged. The funders had no role in the work design, data collection and analysis, or decision and preparation of the manuscript.

## Conflict of Interest Statement

All authors declare that the research was conducted in the absence of any commercial or financial relationships that could be construed as potential conflicts of interest.
